# Effect of sampling time on somatic and germ cell mutations induced by acrylamide in *gpt* delta mice

**DOI:** 10.1186/s41021-021-00175-5

**Published:** 2021-02-17

**Authors:** Soichiro Hagio, Naho Tsuji, Satoshi Furukawa, Kazuya Takeuchi, Seigo Hayashi, Yusuke Kuroda, Masamitsu Honma, Kenichi Masumura

**Affiliations:** 1 Biological Research Laboratories, Nissan Chemical Corporation, 1470 Shiraoka, Shiraoka-shi, Saitama, 349-0294 Japan; 2 Division of Genetics and Mutagenesis, National Institute of Health Sciences, 3-25-26 Tonomachi, Kawasaki-ku, Kawasaki-shi, Kanagawa 210-9501 Japan

**Keywords:** Acrylamide, *gpt* delta transgenic mouse, Germ cell, Mutagenicity

## Abstract

**Background:**

Acrylamide (AA) is a rodent carcinogen and classified by the IARC into Group 2A (probable human carcinogen). AA has been reported to induce mutations in transgenic rodent gene mutation assays (TGR assays), the extent of which is presumed to depend on exposure length and the duration of expression after exposure. In particular, it is not clear in germ cells. To investigate mutagenicity with AA in somatic and germ cells at different sampling times, we conducted TGR assays using *gpt* delta transgenic mice.

**Results:**

The male *gpt* delta mice at 8 weeks of age were treated with AA at 7.5, 15 and 30 mg/kg/day by gavage for 28 days. Peripheral blood was sampled on the last day of the treatment for micronucleus tests and tissues were sampled for gene mutation assays at day 31 and day 77, those being 3 and 49 days after the final treatment (28 + 3d and 28 + 49d), respectively. Another group of mice was treated with *N*-Ethyl-*N*-nitrosourea (ENU) at 50 mg/kg/day by intraperitoneal administration for 5 consecutive days and tissues were sampled at the day 31 and day 77 (5 + 26d and 5 + 72d). Frequencies of micronucleated erythrocytes in the peripheral blood significantly increased at AA doses of 15 and 30 mg/kg/day. Two- to three-fold increases in *gpt* mutation frequencies (MFs) compared to vehicle control were observed in the testes and lung treated with 30 mg/kg/day of AA at both sampling time. In the sperm, the *gpt* MFs and G:C to T:A transversions were significantly increased at 28 + 3d, but not at 28 + 49d. ENU induced *gpt* mutations in these tissues were examined at both 5 + 26d and 5 + 72d. A higher mutant frequency in the ENU-treated sperm was observed at 5 + 72d than that at 5 + 26d.

**Conclusions:**

The *gpt* MFs in the testes, sperm and lung of the AA-treated mice were determined and compared between different sampling times (3 days or 49 days following 28 day-treatment). These results suggest that spermatogonial stem cells are less sensitive to AA mutagenicity under the experimental condition. Prolonged expression time after exposure to AA to detect mutagenicity may be effective in somatic cells but not in germ cells.

**Supplementary Information:**

The online version contains supplementary material available at 10.1186/s41021-021-00175-5.

## Introduction

Acrylamide (AA) has been discovered to be a potent carcinogen in various cooked foods [1–4]. AA can form during processing or with high temperature cooking methods such as flying and baking. AA is also detected in cigarette smoke. Widespread human exposure to AA raizes concerns about public health. The Food Safety Commission of Japan conducted a risk assessment of AA and reported that cancer risk could not be excluded due to the insufficient margins of exposure (MOE), despite there being no clear evidence of effects on human health provided by epidemiological studies [5]. In rodent toxicity studies, major adverse effects were observed by way of neurotoxicity and male reproductive toxicity. Carcinogenicity has been observed in Harderian glands, mammary glands, lung and the forestomach in mice, and in mammary glands, thyroid and testes in rats [4]. Positive results were obtained from in vitro and in vivo genotoxicity studies, although some in vitro studies remain inconclusive because of insufficient metabolic activation in in vitro systems [6–13]. AA is metabolized into active glycidamide (GA) by CYP2E1 [14–17]. GA reacts with DNA and form a number of DNA adducts such as N7-(2-carbamoyl-2-hydroxyethyl)-guanine (N7-GA-Gua), N3-(2-carbamoyl-2-hydroxyethyl)-adenine (N3-GA-Ade) and N1-(2-carbamoyl-2-hydroxyethyl)-adenine (N1-GA-Ade) [18, 19]. It has been proposed that the abasic sites produced by spontaneous depurination of N7-GA-Gua and N3-GA-Ade adducts could lead to G:C to T:A and A:T to T:A transversions [20].

In contrast to somatic tissues, mutagenicity with AA in germ cells is not well investigated. AA induces male reproductive toxicity, and induction of mutations has been observed in testes by treatment of AA in drinking water or i.p. dosing [21–23]. Both positive and negative results have also been reported in induction of chromosomal abberations and micronuclei in spermatogonia, spermatocytse and spermatids, but most of those were i.p. dosing studies [24–29]. Mutations are fixed and accumulate during expression time after dosing in somatic tissues, however, germ cells are different in timing and kinetics of spermatogenesis. Therefore, the timing of exposure and collection of sperm is important to detect mutagenicity in male germ cells [30, 31]. The time for the progression of developing germ cells from exposed spermatogonial stem cells to mature sperm reaching the cauda epididymis is ~ 49 days for the mouse. OECD test guideline TG488 recommennds that sampling of caudal sperm should be conducted only at a minimum of 49 days (mouse) after the end of a 28 day administration period, to assess mutations in spermatogonial stem cells [32]. In this study, mutagenicity with AA in male germ cells exposed at different spermatogenesis stages was investigated. Male *gpt* delta mice were treated with AA for 28 days and *gpt* mutation frequencies (MFs) in the testes, sperm and lung were analyzed at 3 or 49 days after the final treatment (referred to as 28 + 3d and 28 + 49d). Interestingly, *gpt* MFs showed significant increase in the sperm at 28 + 3d, but not at 28 + 49d. These results suggest that AA could be genotoxic with exposure at the later stages of spermatogenesis, and collection of sperm with an expression time of 49 days after exposure may be less sensitive for the detection of germ cell mutagenicity with AA.

## Materials and methods

### Treatments of animals

Male C57BL/6 J *gpt* delta transgenic mice aged 7 weeks were obtained from Japan SLC, Inc. (Shizuoka, Japan). The animals were acclimated for at least 7 days, and administration was initiated at 8 weeks of age when they weighed approximately 22–26 g. The animals were housed in an air-conditioned room under a 12-h light–dark cycle and allowed free access to food and drinking water. The animal experiments in this study were approved by the institutional animal care and use committee and followed recommendations for the handling, maintenance, treatment and sacrificing of the animals.

Acrylamide (AA, CAS No.: 79–06-1) was purchased from Wako Pure Chemical Industries, Ltd. (Osaka, Japan). *N*-Ethyl-*N*-nitrosourea (ENU, CAS No.: 759–73-9) was purchased from Toronto Research Chemicals Inc. (Ontario, Canada). AA was dissolved in sterilized water at appropriate concentrations and administered by oral gavage (10 mL/kg body weight) once a day for 28 consecutive days. ENU was dissolved in saline at appropriate concentration and given by intraperitoneal administration (10 mL/kg body weight) once a day for 5 consecutive days. To set dose levels for AA, a preliminary administration was conducted at doses of 30 and 60 mg/kg/day for 15 days. All animals in the 60 mg/kg dose group were found dead or moribund at up to 15 days. All animals in the 30 mg/kg dose group tolerated AA, and the only finding due to treatment was a hind-leg paralysis. Therefore, the highest dose was set at 30 mg/kg/day for main study. The middle and low doses for AA were set at 15 and 7.5 mg/kg/day. The dose was set at 50 mg/kg/day for ENU because positive results were obtained in the previous report [33]. Negative control animals received sterilized water alone. In the AA treated group and negative control group animals were sacrificed 3 or 49 days after the last administration (referred to as 28 + 3d and 28 + 49d, respectively) (Supplementary Fig. 1). In the ENU treated group, the animals were sacrificed 26 or 72 days after the last administration (as 5 + 26d and 5 + 72d, respectively). Five to six mice per group were used for the experiments. In the AA treated groups, the testes of all dose groups, sperm extracted from cauda epididymis and lung in the highest dose group were used for *gpt* mutation assays. Lung was selected as a somatic tissue because it has been observed as a target of carcinogenicity of AA in mice. Testes, sperm and lung of the ENU-treated and control groups were also used for *gpt* mutation assays.

### Mutation assay and sequencing analysis

The *gpt* mutation assays were conducted as previously described [34]. Briefly, high molecular weight genomic DNA was extracted from the testes and lung using RecoverEase DNA Isolation Kit (Agilent Technologies, Santa Clara, CA). Sperm DNA was extracted as previously described [35]. In brief, cauda epididymis was chopped in 1 mL of phosphate-buffered saline (pH 7.4), mesh-filtered, and pelleted by centrifugation. The pellet was re-suspended in 1× saline sodium citrate (SSC) and 0.15% sodium dodecyl sulfate (SDS). The lysate was centrifuged and the sperm pellet was suspended in 1 mL of 0.2× SSC, 1% SDS, 1 M 2-mercaptoethanol, and 10 mM EDTA (pH 8.0), then digested with 0.5 mg/mL proteinase K at 37 °C overnight. DNA was extracted by phenol/chloroform method. Lambda EG10 phages were rescued from genomic DNA by an in vitro packaging method using Transpack packaging extracts (Agilent Technologies). The phages were incubated with *E.coli* YG6020 and poured onto M9 agar plates containing chloramphenicol (Cm) and 6-thioguanine (6-TG). In order to determine the total number of rescued plasmids, infected cells were also poured onto agar plates containing Cm without 6-TG. The plates were then incubated at 37 °C for selection of 6-TG-resistant colonies, and the *gpt* mutant frequency was calculated by dividing the number of *gpt* mutants by the number of rescued plasmids. A 739-bp DNA fragment containing the 456-bp coding region for the *gpt* gene was amplified by colony-direct PCR and *gpt* mutations were characterized by DNA sequencing with a sequencing primer gptA2 (5′-TCTCGCGCAACCTATTTTCCC-3′). The *gpt* mutation frequency (MF) was calculated by dividing the number of independent *gpt* mutations by the number of rescued plasmids.

### Peripheral blood (PB) MN assay

Blood samples were collected on the last day of AA administration. Approximately 3–10 μl of PB from the mouse tail was placed onto an acridine orange-coated glass slide and covered immediately with a cover glass [36]. The slides were observed by fluorescence microscopy at 600× magnification with B excitation. The frequencies of micronucleated peripheral reticulocytes (MNRETs) were recorded, based on observations of 2000 reticulocytes per animal.

### Statistical analysis

Body and organ weights and *gpt* MF in the testes treated with AA were analyzed statistically using Dunnett’s test or the Steel test. Comparisons of *gpt* MF in sperm and lung between the AA treated groups versus the vehicle control group were analyzed statistically using Student’s or Welch’s t-test. Comparisons of *gpt* MF between the ENU treated group versus the vehicle control group were also analyzed using Student’s or Welch’s t-test. Differences in the incidence of MNRETs in the AA treated groups versus the vehicle control groups were analyzed statistically using Kastenbaum and Bowman’s method [37].

## Results

The *gpt* delta mice were treated with AA by gavage for 28 days and tissues were sampled at 3 and 49 days after final treatment. During treatment, hind-leg paralysis and sluggish movements were observed after 2 weeks from the start of treatment in the 30 mg/kg/day AA-treated group. No significant differences in final body weights for the AA -treated groups were observed. In the 30 mg/kg/day AA-treated group at 3 days after 28 day-treatment, testis weight significantly decreased by 12% below that of controls. No other clinical signs or significant weight changes for the organs were observed (data not shown).

**Fig. 1:**
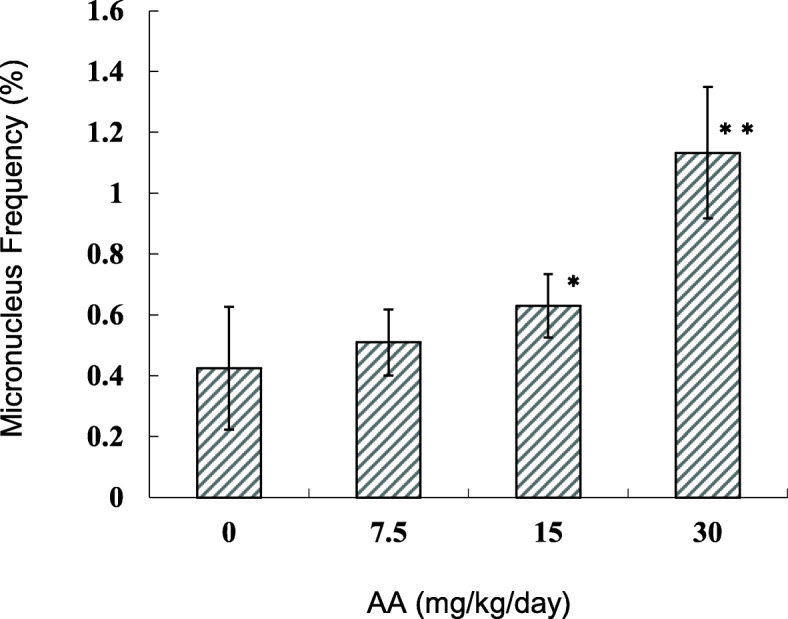
Frequencies of micronucleated peripheral reticulocytes estimated at the last day of AA administration. AA was administered daily by oral gavage for 28 days. The error bar represents the standard deviation. * *P* < 0.05, ** *P* < 0.01, significantly different from vehicle control (Kastenbaum and Bowman’s method)

PB was collected on the last day of AA treatment and MN assays were conducted. The results are shown in Fig. 1. The MNRET frequencies increased in a dose-dependent manner after 28 days of repeated administration with AA and showed a significant increase at doses of 15 and 30 mg/kg/day.

**Fig. 2:**
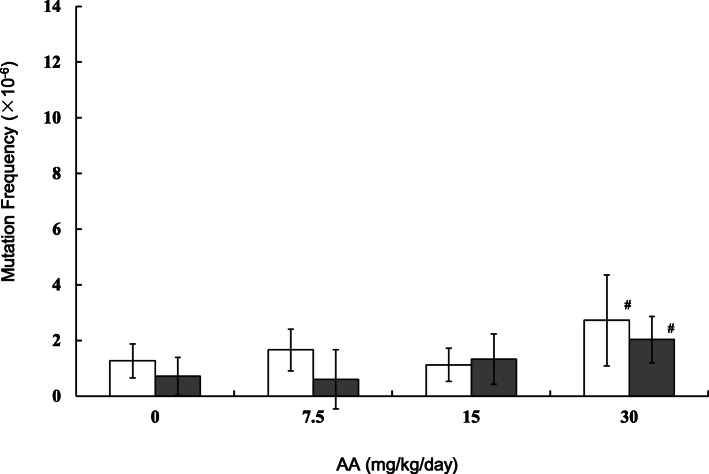
The *gpt* mutation frequencies in the testes treated with AA. The *gpt* MFs were estimated at 3 days (open bars) and 49 days (black bars) after the end of AA administration. AA was administered daily by oral gavage for 28 days. The error bar represents the standard deviation. # P < 0.05, significantly different from vehicle control (Dunnett’s test)

**Fig. 3:**
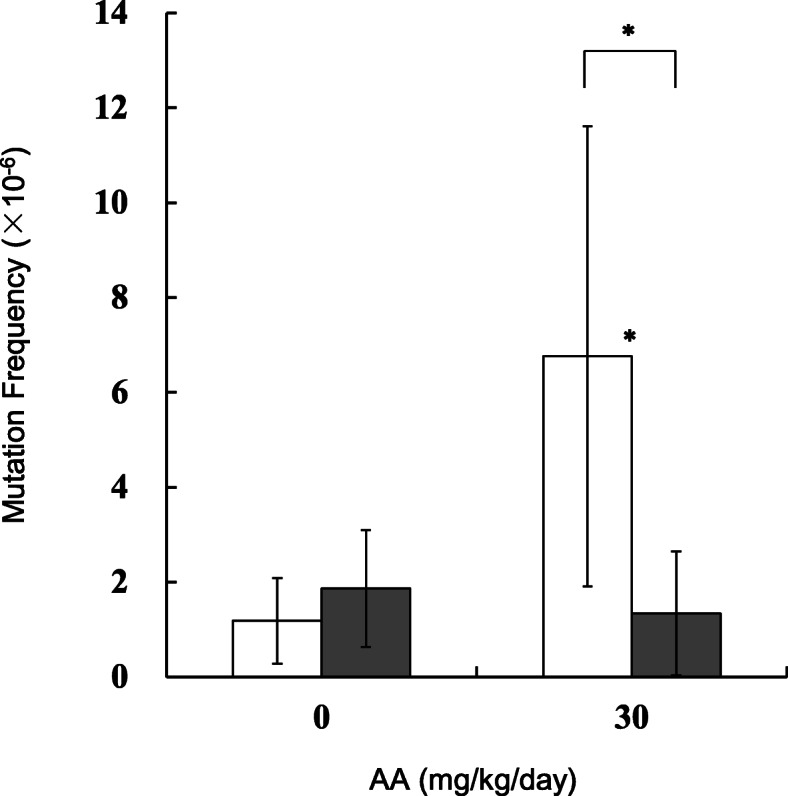
The *gpt* mutation frequencies in the sperm treated with AA. The *gpt* MFs were estimated at 3 days (open bars) and 49 days (black bars) after the end of AA administration. AA was administered daily by oral gavage for 28 days. The error bar represents the standard deviation. * *P* < 0.05, significantly different from vehicle control or between sampling points (Student or Welch t-test)

**Fig. 4:**
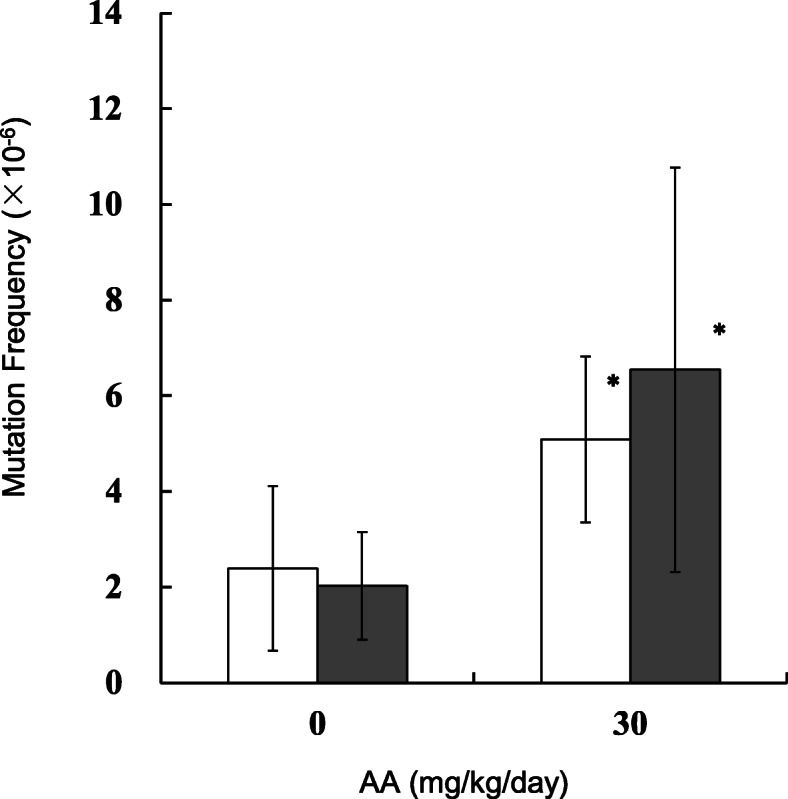
The *gpt* mutation frequencies in the lung treated with AA. The *gpt* MFs were estimated at 3 days (open bars) and 49 days (black bars) after the end of repeated AA administration in mice. AA was administered daily by oral gavage for 28 days. The error bar represents the standard deviation. * *P* < 0.05, significantly different from vehicle control (Student or Welch t-test)

MFs in the testes, sperm and lung of the AA-treated mice were estimated by *gpt* assay. The results are shown in Figs. 2, 3 and 4 (and Supplementary Table 1-3). In the testes, the *gpt* MFs for vehicle control in 28 + 3d and 28 + 49d samples were 1.27 ± 0.61 (× 10^− 6^) and 0.71 ± 0.68 (× 10^− 6^), respectively (Fig. 2). For samples treated with AA at 30 mg/kg/day, the *gpt* MFs were 2.72 ± 1.64 (× 10^− 6^) and 2.03 ± 0.83 (× 10^− 6^) in 28 + 3d and 28 + 49d, respectively. The *gpt* MFs in 30 mg/kg/day AA-treated mice were significantly higher than those of vehicle control. No significant differences between 28 + 3d and 28 + 49d were observed. No significant increase in *gpt* MFs were detected in the groups treated with AA at 7.5 and 15 mg/kg/day. In sperm, the *gpt* MFs for controls at 28 + 3d and 28 + 49d were 1.18 ± 0.91 (× 10^− 6^) and 1.87 ± 1.23 (× 10^− 6^), respectively (Fig. 3). The *gpt* MF in 30 mg/kg/day AA-treated mice was 6.77 ± 4.85 (× 10^− 6^) at 28 + 3d and was significantly higher than that of vehicle controls. On the other hand, no increase in *gpt* MF was observed at 28 + 49d. In the lung, control *gpt* MFs at 28 + 3d and 28 + 49d were 2.40 ± 1.72 (× 10^− 6^) and 2.03 ± 1.12 (× 10^− 6^), respectively. For 28 + 3d and 28 + 49d samples treated with AA at 30 mg/kg/day, the *gpt* MFs were 5.09 ± 1.74 (× 10^− 6^) and 6.54 ± 4.23 (× 10^− 6^), respectively. The *gpt* MFs in 30 mg/kg/day AA-treated mice were significantly about 2 times (28 + 3d) and 3 times (28 + 49d) higher than that of vehicle controls. No significant differences between 3 days and 49 days samples were observed.

To characterize the types of *gpt* mutations induced by AA, the *gpt* mutants obtained were analyzed by DNA sequencing. Mutation spectra are shown in Tables 1, 2 and 3. In the testes, the predominant type of mutation was a G:C to A:T transition in the 30 mg/kg/day AA-treated mice at both 28 + 3d and 28 + 49d, and no significant difference was observed in the mutation spectra (Table 1). In the sperm, the predominant type of mutation was a G:C to A:T transition in the control mice at both 28 + 3d and 28 + 49d. The specific MF of G:C to T:A transversions significantly increased in 28 + 3d samples for the AA-treated mice (Table 2). The specific MFs of G:C to A:T transition and single bp deletions also tend to be higher in the 28 + 3d AA-treated mice. In the lung, the prominent types of mutation in the control mice were G:C to A:T transitions, G:C to T:A transversions and single bp deletions at both 28 + 3d and 28 + 49d (Table 3). Significant increases in the specific MFs of A:T to T:A transversion were observed for both 28 + 3d and 28 + 49d samples from AA-treated mice.

**Table 1:** Mutation spectra of the *gpt* gene in testes of the AA-treated mice

	28 + 3d												28 + 49d											
	0 mg/kg/day			7.5 mg/kg/day			15 mg/kg/day			30 mg/kg/day			0 mg/kg/day			7.5 mg/kg/day			15 mg/kg/day			30 mg/kg/day		
	No.	%	MF (×10^− 6^)	No.	%	MF (×10^− 6^)	No.	%	MF (× 10^− 6^)	No.	%	MF (× 10^− 6^)	No.	%	MF (× 10^− 6^)	No.	%	MF (× 10^− 6^)	No.	%	MF (× 10^− 6^)	No.	%	MF (×10^−6^)
Base substitution																								
Transition																								
G:C to A:T	1	16.7	0.21	6	66.7	1.11	4	44.4	0.50	6	30.0	0.82	1	33.3	0.24	1	33.3	0.20	4	36.4	0.48	8	50.0	1.02
at CpG site	(0)			(2)			(1)			(2)			(0)			(0)			(1)			(6)		
A:T to G:C	0	0.0	0.00	0	0.0	0.00	0	0.0	0.00	1	5.0	0.14	0	0.0	0.00	0	0.0	0.00	0	0.0	0.00	0	0.0	0.00
Transversion																								
G:C to T:A	1	16.7	0.21	0	0.0	0.00	2	22.2	0.25	4	20.0	0.54	1	33.3	0.24	2	66.7	0.40	4	36.4	0.48	2	12.5	0.25
G:C to C:G	1	16.7	0.21	0	0.0	0.00	1	11.1	0.12	2	10.0	0.27	1	33.3	0.24	0	0.0	0.00	0	0.0	0.00	0	0.0	0.00
A:T to T:A	0	0.0	0.00	3	33.3	0.55	0	0.0	0.00	3	15.0	0.41	0	0.0	0.00	0	0.0	0.00	0	0.0	0.00	0	0.0	0.00
A:T to C:G	1	16.7	0.21	0	0.0	0.00	0	0.0	0.00	0	0.0	0.00	0	0.0	0.00	0	0.0	0.00	0	0.0	0.00	0	0.0	0.00
Deletion	2	33.3	0.42	0	0.0	0.00	1	11.1	0.12	3	15.0	0.41	0	0.0	0.00	0	0.0	0.00	3	27.3	0.36	4	25.0	0.51
1 bp	2			0			0			2			0			0			3			4		
> 2 bps	0			0			1			1			0			0			0			0		
Insertion	0	0.0	0.00	0	0.0	0.00	1	11.1	0.12	1	5.0	0.14	0	0.0	0.00	0	0.0	0.00	0	0.0	0.00	2	12.5	0.25
Other	0	0.0	0.00	0	0.0	0.00	0	0.0	0.00	0	0.0	0.00	0	0.0	0.00	0	0.0	0.00	0	0.0	0.00	0	0.0	0.00
Total	6	100	1.27	9	100	1.66	9	100	1.12	20	100	2.72	3	100	0.71	3	100	0.60	11	100	1.33	16	100	2.03

**Table 2:** Mutation spectra of the *gpt* gene in sperm of the AA-treated mice

	28 + 3d						28 + 49d					
	0 mg/kg/day			30 mg/kg/day			0 mg/kg/day			30 mg/kg/day		
	No.	%	MF (×10^−6^)	No.	%	MF (×10^−6^)	No.	%	MF (×10^−6^)	No.	%	MF (×10^−6^)
Base substitution												
Transition												
G:C to A:T	4	50.0	0.59	7	31.8	2.15	8	72.7	1.36	6	50.0	0.67
at CpG site	(2)			(5)			(4)			(4)		
A:T to G:C	0	0.0	0.00	1	4.5	0.31	1	9.1	0.17	1	8.3	0.11
Transversion												
G:C to T:A	1	12.5	0.15	6	27.3	1.85^*^	0	0.0	0.00	2	16.7	0.22
G:C to C:G	0	0.0	0.00	1	4.5	0.31	0	0.0	0.00	0	0.0	0.00
A:T to T:A	1	12.5	0.15	1	4.5	0.31	0	0.0	0.00	1	8.3	0.11
A:T to C:G	0	0.0	0.00	1	4.5	0.31	0	0.0	0.00	0	0.0	0.00
Deletion	2	25.0	0.30	4	18.2	1.23	2	18.2	0.34	2	16.7	0.22
1 bp	1			3			2			1		
> 2 bps	1			1			0			1		
Insertion	0	0.0	0.00	0	0.0	0.00	0	0.0	0.00	0	0.0	0.00
Other	0	0.0	0.00	1	4.5	0.31	0	0.0	0.00	0	0.0	0.00
Total	8	100	1.18	22	100	6.77	11	100	1.87	12	100	1.34

*p < 0.05 vs control

**Table 3:** Mutation spectra of the *gpt* gene in lung of the AA-treated mice

	28 + 3d						28 + 49d					
	0 mg/kg/day			30 mg/kg/day			0 mg/kg/day			30 mg/kg/day		
	No.	%	MF (×10^−6^)	No.	%	MF (×10^−6^)	No.	%	MF (×10^−6^)	No.	%	MF (×10^−6^)
Base substitution												
Transition												
G:C to A:T	6	27.3	0.65	10	30.3	1.54	7	46.7	0.95	14	33.3	2.18
at CpG site	(5)			(8)			(4)			(7)		
A:T to G:C	1	4.5	0.11	1	3.0	0.15	1	6.7	0.14	7	16.7	1.09
Transversion												
G:C to T:A	7	31.8	0.76	3	9.1	0.46	3	20.0	0.41	4	9.5	0.62
G:C to C:G	1	4.5	0.11	0	0.0	0.00	0	0.0	0.00	2	4.8	0.31
A:T to T:A	0	0.0	0.00	5	15.2	0.77^*^	0	0.0	0.00	8	19.0	1.25^*^
A:T to C:G	1	4.5	0.11	3	9.1	0.46	0	0.0	0.00	1	2.4	0.16
Deletion	6	27.3	0.65	10	30.3	1.54	4	26.7	0.54	5	11.9	0.78
1 bp	5			10			3			5		
> 2 bps	1			0			1			0		
Insertion	0	0.0	0.00	0	0.0	0.00	0	0.0	0.00	0	0.0	0.00
Other	0	0.0	0.00	1	3.0	0.15	0	0.0	0.00	1	2.4	0.16
Total	22	100	2.40	33	100	5.09	15	100	2.03	42	100	6.54

*Significantly different from control (*p* < 0.05)

**Fig. 5:**
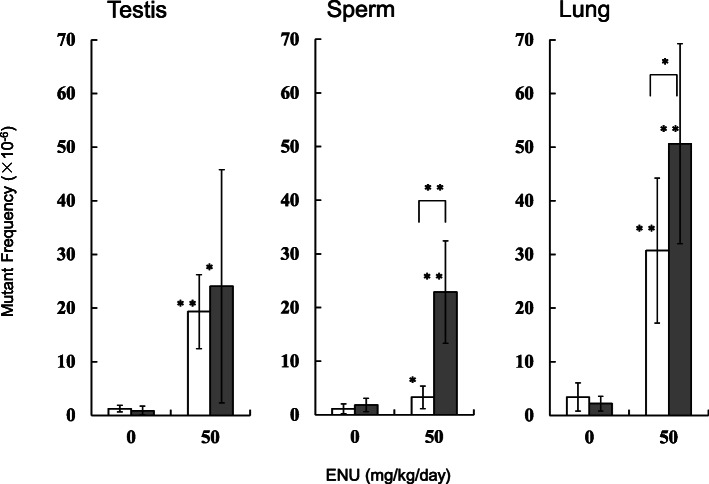
The *gpt* mutant frequencies in the testes, sperm and lung treated with ENU. ENU was administered daily by intraperitoneal administration for 5 days and the *gpt* MFs were estimated at at day 31 (5 + 26d) (open bars) and day 77 (5 + 72d) (dark bars). For ENU-treated groups, mutant frequency is shown, instead of mutation frequency. The error bar represents the standard deviation. * *P* < 0.05, ** *P* < 0.01, significantly different from vehicle control or between sampling points (Student or Welch t-test)

Another group of mice was treated with ENU by intraperitoneal administration for 5 days and tissues were sampled after 26 days (day 31) and 72 days (day 77) of final treatment. The weights of testes in the ENU-treated mice were significantly decreased by 65% at day 31 (5 + 26d) and by 38% at day 77 (5 + 72d), compared with the controls. Weights of epididymides were significantly decreased by 32% at 5 + 26d and by 14% at 5 + 72d, compared with the controls. No significant differences in final body weights between control and ENU-treated groups were observed at either 5 + 26d or 5 + 72d (data not shown). The *gpt* mutant frequencies in the testes, sperm and lung of the ENU-treated mice are shown in Fig. 5 (and Supplementary Table 1-3). The *gpt* mutant frequencies in the testes of the ENU-treated mice were significantly higher than that of vehicle controls, but no significant difference between 5 + 26d and 5 + 72d was observed. In the sperm, the *gpt* mutant frequencies were significantly higher than those of controls at both 5 + 26d and 5 + 72d, however, the mutant frequency was 7-times higher at 5 + 72d than that at 5 + 26d. In the lung, the *gpt* mutant frequencies in ENU-treated mice were significantly higher than that of controls. The *gpt* mutant frequency in the ENU-treated group at 5 + 72d was significantly 1.6-fold higher than that at 5 + 26d.

## Discussion

Many rodent studies for genotoxicity and carcinogenicity with AA have employed administration in drinking water [9, 12, 13, 21] . It is known that higher AA doses in drinking water cause decreased water consumption. In contrast, in this study, the mice were administered AA by gavage daily, for 28 days. In the highest dose, 30 mg/kg/day AA-treated group, toxic effects such as hind-leg paralysis and sluggish movement were observed 2 weeks after the beginning of treatment, and testis weight loss was observed at 3 days after final treatment. This bespoke neurotoxicity and reproductive toxicity for AA, and AA at 30 mg/kg/day was close to the maximum tolerable dose (MTD) under this experimental condition. When AA was administrered in drinking water in mice, similar toxicity was reported at doses of 400 ppm (59 mg/kg/day) or 7 mM (500 ppm, 88–98 mg/kg/day) [13, 21].

PB MN assays at day 28 showed dose-dependent increases of %MNRET, and a significant increase was observed at doses of 15 and 30 mg/kg/day (Fig. 1). Positive response of the PB MN may suggest that systemic exposure of AA and its metabolite caused genotoxicity such as chromosome aberrations. It has been reported that MN assays using flow cytometry methods in B6C3F1 mice resulted in significant MN induction at doses of more than 6 mg/kg/day of AA [9, 38]. This may suggest that sensitivity to MN induction by AA in C57BL/6 J mice was relatively subdued as compared B6C3F1 background.

MFs in the testes, sperm and lung of the AA-treated mice were estimated, and two different sampling points (3 or 49 days after final treatment) were compared (Figs. 2, 3 and 4). In the lung and testes of the 30 mg/kg/day AA-treated group, the *gpt* MFs were 2–3 times higher than those of vehicle controls and no significant difference between 28 + 3d and 28 + 49d was observed. This suggests that 28 + 3d could be sufficient time for fixation of AA-induced mutations in those tissues. Although mutation can accumulate with repeated dosing, both the MN in PB and the *gpt* MF in lung increased 2–3 times higher than control at a dose of 30 mg/kg/day. It may suggest different sensitivity of chromosomal aberrations and gene mutations. Interestingly, the *gpt* MF in the sperm of the 30 mg/kg/day of AA-treated group was significantly 6 times higher than that of control at 28 + 3d, but no increase of the *gpt* MF was observed at 28 + 49d. This may suggest different sensitivity at the AA-exposed spermatogenesis stages. Russell reported an influence of exposed germ-cell stages on test effectiveness with various mutagens analyzed by mouse specific locus test (SLT), and AA-induced mutations are the most sensitive with exposure at post-spermatogonial stages [39]. It has been suggested that the latter stage of spermatogenesis lacks in DNA replication and cell proliferation, and undergoes progressive loss of DNA repair capacity [31, 40]. If that is the case, then DNA damages induced by AA exposure might not be fixed as mutations at this stage. One possibility is that AA-induced DNA damages remaining in the sperm could be transferred into zygotes, then fixed as mutations by cell divisions with DNA replication. In fact, genomic DNAs extracted from 28 + 3d sperm seemed to be substantially damaged because their in vitro packaging efficiency was lower than that in control groups (data not shown). It is not clear if the AA-damaged DNA could cause ex vivo mutations in *E. coli* cells in the *gpt* assay. However, a positive selection mechanism in transgenic rodent gene mutation assays such as *gpt* assay could make grow only fixed mutants in the selection plates, and suppress ex vivo mutations [41, 42]. Why was the higher MF observed in the 28 + 3d sperm? One possibility is that testicular toxicity might affect sample preparation and contaminate testicular somatic cells. But the MF for the 28 + 3d sperm samples was higher than that in the testes of the same AA-treated group. Another possible explanation is that AA is more sensitive on mutagenesis at the post-spermatogonial stage than that in spermatogonial stem cells. The time for progression of mouse germ cells from spermatogonial stem cells to mature sperm reaching the cauda epididymis is ~ 49 days [30]. In the 28 + 3d sperm, germ cells might have been partially exposed in the stages of differentiating spermatogonia and meiosis, with DNA repair causing mutagenesis. On the other hand, the result with 28 + 49d sperm suggested that AA may not induce mutagenicity in spermatogonial stem cells under our experimental conditions. AA-induced DNA adducts might have been rapidly removed or repaired in the stem cells. To elucidate why AA exposure at the post-spermatogonial stage resulted in higher MF in the sperm DNA, more studies such as analyses of DNA adducts in the AA-exposed germ cells and effects of toxicity on spermatogenesis are necessary.

G:C to T:A transversions were significantly increased in the 28 + 3d sperm of the AA-treated mice (Table 2). In the lung of the AA-treated mice, A:T to T:A transversions were significantly increased at both 28 + 3d and 28 + 49d (Table 3). Because of small increase of the MF and limited number of sequenced mutants, no significant increase of specific type of mutations was observed in the testes. G:C to A:T transitions commonly contributed to the higher MF observed in the testes, sperm and lung of the AA-treated mice. N7-GA-Gua and N3-GA-Ade adducts induced by AA exposure are depurinating adducts resulting in apurinic sites. Replication leads to incorporate deoxyadenosine oppositing to abasic sites, and causes the G:C to T:A and A:T to T:A transversions, respectively. N1-GA-Ade adducts could lead to the induction of G:C to A:T transitions [13, 19, 20, 43, 44]. Mutation spectra in those tissues may suggest different contribution of the specific DNA adducts.

ENU induced *gpt* mutations in the testes, sperm and lung were examined (Fig. 5). Although the mutant frequencies in the ENU-treated sperm were significantly higher at both 5 + 26d and 5 + 72d, the mutant frequency was 7 times higher at 5 + 72d than that at 5 + 26d. Analyses by SLT have suggested that ENU-induced mutations are the most sensitive to exposure at the spermatogonial stage [39]. Because it takes ~ 49 days to go from spermatogonial stem cells to mature sperm, day 31 (5 days dosing followed by 26 days expression time) is not long enough to take this period of spermatogenesis into account. Our results supported that 28 + 3d sperm is less sensitive for the detection of mutagenicity with ENU in male germ cells [31]. In the lung, a higher mutant frequency was observed at 5 + 72d than that at 5 + 26d. This may suggest that longer expression time can fix more mutations in somatic tissues having relatively slow cell-proliferation.

In this study, mutagenicity with AA in male germ cells exposed at different spermatogenesis stages was investigated. The results suggest that 28 + 49d sperm, which is exposed to AA at the spermatogonial stem cell stage, does not present with an increase in MF. On the other hand, the 28 + 3d sperm, which was exposed to AA at the post-spermatogonial stage, resulted in higher MF. In contrast, longer expression time resulted in higher mutant frequency in the sperm of the ENU-treated mice. Sensitive sampling points for detecting germ cell mutagenicity could be different for different mutagens. The difference in a critical window between AA and ENU maybe caused by a difference in mode-of-action. AA needs metabolic activation for mutagenesis but ENU can alkylate DNA without metabolic activation. Efficiency of DNA repair could be different for mutagen-specific DNA adducts. Recommended regimens for the analysis of mutations in germ cells was updated in OECD test guideline TG488 in 2020 [32]. The guideline suggests that collection of germ cells from the seminiferous tubules, a mixed population of spermatogonia, spermatocytes and spermatids, at a sampling time longer than 3 days after administration for 28 days is better for the assessment of germ cell mutagenicity, and that a 28 + 28d regimen enables the evaluation of mutations in a majority of germ cell populations exposed during the proliferative phase of spermatogenesis. Sampling cell populations covering different spermatogenesis stages may contribute to more comprehensive assays for the detection of germ cell mutagenicity.

Further study is necessary to elucidate genotoxic effects of chronically exposed AA in germlines and subsequent generations. Recent advances in sequencing technology has been able to detect de novo mutations induced in the offspring of mutagen-treated parents [35, 45, 46]. It is important to investigate whether dietary AA intake could induced DNA damage and results in germline mutations and heritable effects.

## Conclusions

The MFs in the testes, sperm and lung of the AA-treated *gpt* delta mice were examined in different sampling times after dosing for 28 days. These results suggested that spermatogonial stem cells are less sensitive to AA mutagenicity under the experimental condition. Prolonged expression time after exposure to AA to detect mutagenicity may be effective in somatic cells but sensitive sampling points for detecting germ cell mutagenicity could be different for different mutagens.

## Supplementary Information

**Additional file 1: Supplementary Fig. 1**. Experimental design

**Additional file 2: Supplementary Table 1**. The gpt mutation frequencies in the testis of the AA or ENU-treated mice. **Supplementary Table 2**. The gpt mutation frequencies in the sperm of the AA or ENU-treated mice. **Supplementary Table 3**. The gpt mutation frequencies in the lung of the AA or ENU-treated mice.

## Data Availability

All data generated or analysed during this study are included in this published article and its supplementary information files.

## Abbreviations

6-TG: 6-thioguanine
AA: Acrylamide
Cm: Chloramphenicol
ENU: *N*-ethyl-*N*-nitrosourea
GA: Grycidamide
IARC: International Agency for Research on Cancer
MF: Mutation frequenc
MN: Micronucleus
MNRET: Micronucleated peripheral reticulocyte
MOE: Margins of exposure
MTD: Maximum tolerable dose
N1-GA-Ade: N1-(2-carbamoyl-2-hydroxyethyl)-adenine
N3-GA-Ade: N3-(2-carbamoyl-2-hydroxyethyl)-adenine
N7-GA-Gua: N7-(2-carbamoyl-2-hydroxyethyl)-guanine
PB: Peripheral blood
SLT: Specific locus test
TGR: Transgenic rodent gene mutaion assay

## References

[1] Rosen J, Hellenas KE (2002). Analysis of acrylamide in cooked foods by liquid chromatography tandem mass spectrometry. Analyst..

[2] Tareke E, Rydberg P, Karlsson P, Eriksson S, Tornqvist M (2002). Analysis of acrylamide, a carcinogen formed in heated foodstuffs. J Agric Food Chem.

[3] Mucci LA, Wilson KM (2008). Acrylamide intake through diet and human cancer risk. J Agric Food Chem.

[4] National Toxicology P (2012). Toxicology and carcinogenesis studies of acrylamide (CASRN 79-06-1) in F344/N rats and B6C3F1 mice (feed and drinking water studies). Natl Toxicol Program Tech Rep Ser.

[5] Food Safety Commission of J (2016). Acrylamide in Foods Generated through Heating (Contaminants). Food Saf (Tokyo).

[6] Dearfield KL, Douglas GR, Ehling UH, Moore MM, Sega GA, Brusick DJ (1995). Acrylamide: a review of its genotoxicity and an assessment of heritable genetic risk. Mutat Res.

[7] Dearfield KL, Abernathy CO, Ottley MS, Brantner JH, Hayes PF (1988). Acrylamide: its metabolism, developmental and reproductive effects, genotoxicity, and carcinogenicity. Mutat Res.

[8] Abramsson-Zetterberg L (2003). The dose-response relationship at very low doses of acrylamide is linear in the flow cytometer-based mouse micronucleus assay. Mutat Res.

[9] Hobbs CA, Davis J, Shepard K, Chepelev N, Friedman M, Marroni D (2016). Differential genotoxicity of acrylamide in the micronucleus and *Pig-a* gene mutation assays in F344 rats and B6C3F1 mice. Mutagenesis..

[10] Koyama N, Yasui M, Oda Y, Suzuki S, Satoh T, Suzuki T (2011). Genotoxicity of acrylamide *in vitro*: acrylamide is not metabolically activated in standard *in vitro* systems. Environ Mol Mutagen.

[11] Manjanatha MG, Aidoo A, Shelton SD, Bishop ME, McDaniel LP, Lyn-Cook LE (2006). Genotoxicity of acrylamide and its metabolite glycidamide administered in drinking water to male and female big blue mice. Environ Mol Mutagen.

[12] Manjanatha MG, Guo LW, Shelton SD, Doerge DR (2015). Acrylamide-induced carcinogenicity in mouse lung involves mutagenicity: *cII* gene mutations in the lung of big blue mice exposed to acrylamide and glycidamide for up to 4 weeks. Environ Mol Mutagen.

[13] Ishii Y, Matsushita K, Kuroda K, Yokoo Y, Kijima A, Takasu S (2015). Acrylamide induces specific DNA adduct formation and gene mutations in a carcinogenic target site, the mouse lung. Mutagenesis..

[14] Calleman CJ, Bergmark E, Costa LG (1990). Acrylamide is metabolized to glycidamide in the rat: evidence from hemoglobin adduct formation. Chem Res Toxicol.

[15] Sumner SC, Fennell TR, Moore TA, Chanas B, Gonzalez F, Ghanayem BI (1999). Role of cytochrome P450 2E1 in the metabolism of acrylamide and acrylonitrile in mice. Chem Res Toxicol.

[16] Ghanayem BI, McDaniel LP, Churchwell MI, Twaddle NC, Snyder R, Fennell TR (2005). Role of CYP2E1 in the epoxidation of acrylamide to glycidamide and formation of DNA and hemoglobin adducts. Toxicol Sci.

[17] Fennell TR, Snyder RW, Krol WL, Sumner SC (2003). Comparison of the hemoglobin adducts formed by administration of *N*-methylolacrylamide and acrylamide to rats. Toxicol Sci.

[18] Segerback D, Calleman CJ, Schroeder JL, Costa LG, Faustman EM (1995). Formation of N-7-(2-carbamoyl-2-hydroxyethyl)guanine in DNA of the mouse and the rat following intraperitoneal administration of [14C]acrylamide. Carcinogenesis..

[19] Doerge DR, Gamboa da Costa G, McDaniel LP, Churchwell MI, Twaddle NC, Beland FA (2005). DNA adducts derived from administration of acrylamide and glycidamide to mice and rats. Mutat Res.

[20] Randall SK, Eritja R, Kaplan BE, Petruska J, Goodman MF (1987). Nucleotide insertion kinetics opposite abasic lesions in DNA. J Biol Chem.

[21] Wang RS, McDaniel LP, Manjanatha MG, Shelton SD, Doerge DR, Mei N (2010). Mutagenicity of acrylamide and glycidamide in the testes of big blue mice. Toxicol Sci.

[22] Koyama N, Yasui M, Kimura A, Takami S, Suzuki T, Masumura K (2011). Acrylamide genotoxicity in young versus adult *gpt* delta male rats. Mutagenesis..

[23] Mei N, McDaniel LP, Dobrovolsky VN, Guo X, Shaddock JG, Mittelstaedt RA (2010). The genotoxicity of acrylamide and glycidamide in big blue rats. Toxicol Sci.

[24] Shiraishi Y (1978). Chromosome aberrations induced by monomeric acrylamide in bone marrow and germ cells of mice. Mutat Res.

[25] Backer LC, Dearfield KL, Erexson GL, Campbell JA, Westbrook-Collins B, Allen JW (1989). The effects of acrylamide on mouse germ-line and somatic cell chromosomes. Environ Mol Mutagen.

[26] Collins BW, Howard DR, Allen JW (1992). Kinetochore-staining of spermatid micronuclei: studies of mice treated with X-radiation or acrylamide. Mutat Res.

[27] Russo A, Gabbani G, Dorigo E (1994). Evaluation of sister-chromatid exchanges in mouse spermatogonia: a comparison between the classical fluorescence plus Giemsa staining and an immunocytochemical approach. Mutat Res.

[28] Xiao Y, Tates AD (1994). Increased frequencies of micronuclei in early spermatids of rats following exposure of young primary spermatocytes to acrylamide. Mutat Res.

[29] Lahdetie J, Suutari A, Sjoblom T (1994). The spermatid micronucleus test with the dissection technique detects the germ cell mutagenicity of acrylamide in rat meiotic cells. Mutat Res.

[30] Marchetti F, Aardema M, Beevers C, van Benthem J, Douglas GR, Godschalk R (2018). Simulation of mouse and rat spermatogenesis to inform genotoxicity testing using OECD test guideline 488. Mutat Res Genet Toxicol Environ Mutagen.

[31] Marchetti F, Aardema MJ, Beevers C, van Benthem J, Godschalk R, Williams A (2018). Identifying germ cell mutagens using OECD test guideline 488 (transgenic rodent somatic and germ cell gene mutation assays) and integration with somatic cell testing. Mutat Res Genet Toxicol Environ Mutagen.

[32] OECD. Test No. 488: Transgenic Rodent Somatic and Germ Cell Gene Mutation Assays. OECD Guidelines for the Testing of Chemicals, Section 4. Paris: OECD Publishing; 2020. 10.1787/9789264203907-en.

[33] Douglas GR, Jiao J, Gingerich JD, Gossen JA, Soper LM (1995). Temporal and molecular characteristics of mutations induced by ethylnitrosourea in germ cells isolated from seminiferous tubules and in spermatozoa of *lacZ* transgenic mice. Proc Natl Acad Sci U S A.

[34] Nohmi T, Suzuki T, Masumura K (2000). Recent advances in the protocols of transgenic mouse mutation assays. Mutat Res.

[35] Masumura K, Toyoda-Hokaiwado N, Ukai A, Gondo Y, Honma M, Nohmi T (2016). Estimation of the frequency of inherited germline mutations by whole exome sequencing in ethyl nitrosourea-treated and untreated *gpt* delta mice. Genes Environ.

[36] Hayashi M, Morita T, Kodama Y, Sofuni T, Ishidate M (1990). The micronucleus assay with mouse peripheral blood reticulocytes using acridine orange-coated slides. Mutat Res.

[37] Kastenbaum MA, Bowman KO (1970). Tables for determining the statistical significance of mutation frequencies. Mutat Res.

[38] Zeiger E, Recio L, Fennell TR, Haseman JK, Snyder RW, Friedman M (2009). Investigation of the low-dose response in the *in vivo* induction of micronuclei and adducts by acrylamide. Toxicol Sci.

[39] Russell LB (2004). Effects of male germ-cell stage on the frequency, nature, and spectrum of induced specific-locus mutations in the mouse. Genetica..

[40] Olsen AK, Lindeman B, Wiger R, Duale N, Brunborg G (2005). How do male germ cells handle DNA damage?. Toxicol Appl Pharmacol.

[41] Nohmi T, Katoh M, Suzuki H, Matsui M, Yamada M, Watanabe M (1996). A new transgenic mouse mutagenesis test system using Spi- and 6-thioguanine selections. Environ Mol Mutagen.

[42] Lambert IB, Singer TM, Boucher SE, Douglas GR (2005). Detailed review of transgenic rodent mutation assays. Mutat Res.

[43] Zhivagui M, Ng AWT, Ardin M, Churchwell MI, Pandey M, Renard C (2019). Experimental and pan-cancer genome analyses reveal widespread contribution of acrylamide exposure to carcinogenesis in humans. Genome Res.

[44] Gamboa da Costa G, Churchwell MI, Hamilton LP, Von Tungeln LS, Beland FA, Marques MM (2003). DNA adduct formation from acrylamide via conversion to glycidamide in adult and neonatal mice. Chem Res Toxicol.

[45] Masumura K, Toyoda-Hokaiwado N, Ukai A, Gondo Y, Honma M, Nohmi T (2016). Dose-dependent de novo germline mutations detected by whole-exome sequencing in progeny of ENU-treated male *gpt* delta mice. Mutat Res.

[46] Satoh Y, Asakawa JI, Nishimura M, Kuo T, Shinkai N, Cullings HM (2020). Characteristics of induced mutations in offspring derived from irradiated mouse spermatogonia and mature oocytes. Sci Rep.

