# Supplementary figures and images for: Effect of sampling time on somatic and germ cell mutations induced by acrylamide in gpt delta mice
Source: Genes Environ. 2021 Feb 17;43:4. doi: 10.1186/s41021-021-00175-5 (PMC7890838; doi:10.1186/s41021-021-00175-5)

Supplementary fig. 1: experimental design

Animals: male C57BL/6J *gpt* delta mice, 8 week-old, 5~6 mice/group

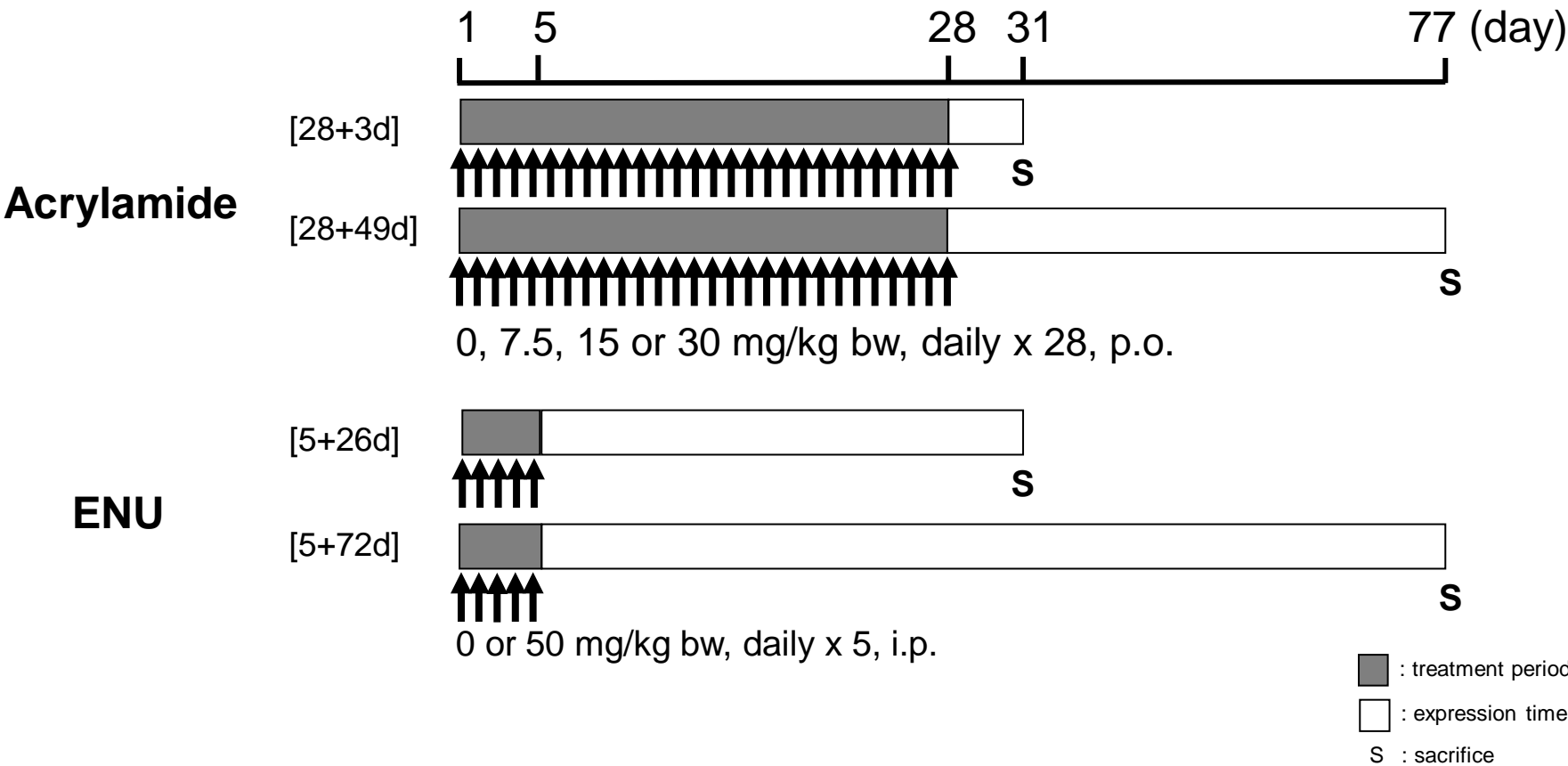

Supplement: Supplementary file 1 — Additional file 1: Supplementary Fig. 1. Experimental design [file 41021_2021_175_MOESM1_ESM.pdf]
